# Thermogel-Coated Poly(ε-Caprolactone) Composite Scaffold for Enhanced Cartilage Tissue Engineering

**DOI:** 10.3390/polym8050200

**Published:** 2016-05-19

**Authors:** Shao-Jie Wang, Zheng-Zheng Zhang, Dong Jiang, Yan-Song Qi, Hai-Jun Wang, Ji-Ying Zhang, Jian-Xun Ding, Jia-Kuo Yu

**Affiliations:** 1 Institute of Sports Medicine, Beijing Key Laboratory of Sports Injuries, Peking University Third Hospital, Beijing 100191, China; wade301@163.com (S.-J.W.); zzz1985114@163.com (Z.-Z.Z.); bysyjiangdong@126.com (D.J.); malaqinfu@126.com (Y.-S.Q.); sportsmedwang@163.com (H.-J.W.); zjychh@163.com (J.-Y.Z.); 2 Key Laboratory of Polymer Ecomaterials, Changchun Institute of Applied Chemistry, Chinese Academy of Sciences, Changchun 130022, China

**Keywords:** thermogel, coating, scaffold, mesenchymal stromal cells, cartilage regeneration

## Abstract

A three-dimensional (3D) composite scaffold was prepared for enhanced cartilage tissue engineering, which was composed of a poly(ε-caprolactone) (PCL) backbone network and a poly(lactide-*co*-glycolide)-*block*-poly(ethylene glycol)-*block*-poly(lactide-*co*-glycolide) (PLGA–PEG–PLGA) thermogel surface. The composite scaffold not only possessed adequate mechanical strength similar to native osteochondral tissue as a benefit of the PCL backbone, but also maintained cell-friendly microenvironment of the hydrogel. The PCL network with homogeneously-controlled pore size and total pore interconnectivity was fabricated by fused deposition modeling (FDM), and was impregnated into the PLGA–PEG–PLGA solution at low temperature (e.g., 4 °C). The PCL/Gel composite scaffold was obtained after gelation induced by incubation at body temperature (*i.e.*, 37 °C). The composite scaffold showed a greater number of cell retention and proliferation in comparison to the PCL platform. In addition, the composite scaffold promoted the encapsulated mesenchymal stromal cells (MSCs) to differentiate chondrogenically with a greater amount of cartilage-specific matrix production compared to the PCL scaffold or thermogel. Therefore, the 3D PCL/Gel composite scaffold may exhibit great potential for *in vivo* cartilage regeneration.

## 1. Introduction

Articular cartilage defects may arise from either acute trauma or repetitive injury [1]. The limited intrinsic healing capacity of cartilage necessitates the development of techniques to repair cartilage defects. The strategies of cartilage repair are mainly comprised of marrow stimulation techniques, mosaicplasty, and chondrocyte-related tissue engineering. In the past two decades, cells-based therapy has evolved to mesenchymal stromal cells (MSCs)-incorporated cartilage repair that might enable us to provide a one-step solution with promising outcomes [2].

Polymers, such as polylactide (PLA), polyglycolide (PGA), and their copolymers (PLGA), and poly(ε-caprolactone) (PCL), are the main synthetic scaffold materials, which have been fabricated into various forms, such as hydrogels and porous sponges for cell delivery in cartilage regeneration [3]. Among these materials, PCL has been shown to have good mechanical properties, and can maintain phenotype, and promote chondrocyte proliferation [4]. Although the porous polymer scaffold can provide superior mechanical support, previous research has demonstrated that most cells tended to adhere and migrate only on the surfaces of pores [5].

Hydrogels are water swellable, yet water insoluble, crosslinked networks, and suitable for the delivery of cells and bioactive agents. Additionally, hydrogels are of high water contents, which facilitate the transport of nutrients and waste, as well as maintain a homogenous cell suspension. The encapsulated cells typically display a rounded cell morphology that may induce a chondrocyte phenotype [6]. In recent years, the injectable thermogels formed by the sol–gel phase transition have been recently used in many biomedical fields, such as drug delivery, tissue engineering, wound healing, and cell therapy [7]. Poly(lactide-*co*-glycolide)–poly(ethylene glycol)–poly(lactide-*co*-glycolide) (PLGA–PEG–PLGA) triblock copolymer has been proven to be a potential matrix of thermogel, which shows the minimal invasive way of delivering cells and bioactive molecules. PLGA–PEG–PLGA copolymer dissolves in water at low temperature (e.g., 4 °C), while the solution gels spontaneously under body temperature (*i.e.*, 37 °C) [8,9,10]. However, the major limitation of thermo-sensitive hydrogels is their unsatisfactory mechanical property, which hinders their further application in a mechanically challenging microenvironment, such as knee joints. The combined system of hydrogel reinforced by a porous scaffold has been suggested to exhibit the mechanical stability and biomimetic extracellular microenvironment [3].

Fused deposition modeling (FDM) has recently gained the popularity of biomaterial researchers because this material processing technique allows for building of scaffolds with highly regular morphology and completely interconnected pores and channels. These scaffold features could not be achieved by conventional scaffold formation methods, such as fiber-bonding, solvent casting and particulate leaching, or membrane lamination [11].

**Figure 1:**
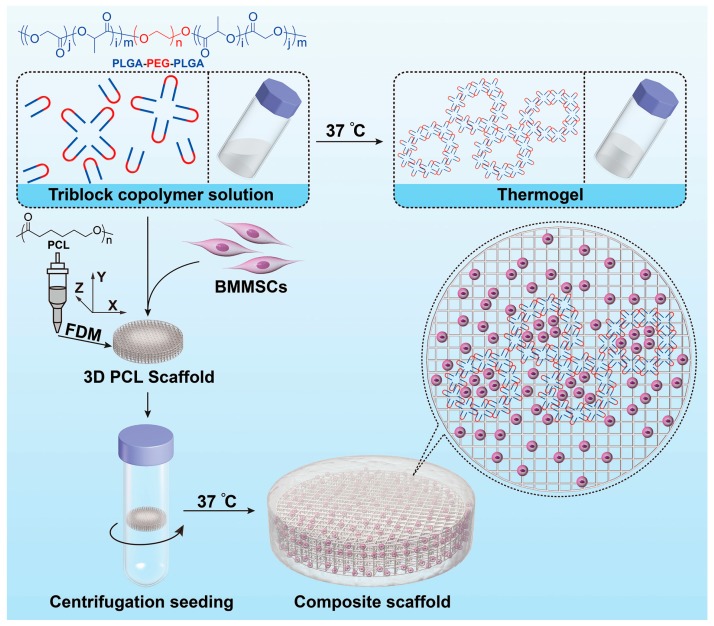
Schematic illustration for fabrication of PCL/Gel composite scaffold as matrix of BMMSCs.

In the current work, the three-dimensional (3D) PCL scaffold was integrated with the PLGA–PEG–PLGA thermogel to form a composite scaffold for promising cartilage tissue engineering (Figure 1). The 3D porous PCL backbone network was fabricated by FDM, and the PLGA–PEG–PLGA thermogel was coated onto the surface through solution infiltration and temperature increase. Bone marrow MSCs (BMMSCs) were seeded onto the PCL/Gel composite scaffold, or single thermogel or PCL scaffold. The aim of this study was to compare the proliferation, survival, and chondrogenic capacities of BMMSCs in thermogel, PCL scaffold, and PCL/Gel scaffold *in vitro*. The MSCs/PCL/Gel construct demonstrated the best efficacy of chondrogenic capacity and extracellular matrix (ECM) production with excellent mechanical property and appropriate microenvironment.

## 2. Materials and Methods

### 2.1. Fabrication of PCL Scaffold

Medical grade PCL (number-average molecular weight (*M*_n_) = 74,600 g·mol^−1^, melting point (MP) = 52.9 °C) was provided by Changchun SinoBiomaterials Co., Ltd. (Changchun, China). PCL was melted in the extrusion head reservoir at a temperature of 130 °C, pressurized at 800 kPa, and extruded to produce a melt filament through a heated metal micronozzle (gauge 21, I.D. = 510 μm). The movement of micronozzle along the X, Y, and Z axes were realized with computer-aided manufacturing software (Delta Tau Data Systems Inc., Chatsworth, CA, USA). The nozzle speed was set at 0.88 mm·s^−1^. The fibers were well aligned and fused at the 0°- to 90°- oriented junctions with fiber spacing of 300 μm and a Z axis interlayer increment of 300 μm.

### 2.2. Micro-Morphology and Pore Size of PCL Scaffold

The surface morphology of scaffold was observed under scanning electron microscopy (SEM). The critical point drying was performed in liquid carbon dioxide (CO_2_) at 37 °C. The samples were vacuum-coated with a 5-nm layer of gold in a high-vacuum gold sputter coater and observed by a JSM5600LV SEM (JEOL USA, Inc., Peabody, MA, USA). Pore size was measured by using Image-pro Plus software 6.0 (Media Cybernetics, Silver Spring, MD, USA). Ten pores were measured per scaffold, and three scaffolds were examined.

### 2.3. Thermogel Preparation

PEG (*M*_n_ = 1000 g·mol^−1^) and stannous octoate (Sn(Oct)_2_) were purchased from Sigma-Aldrich (Shanghai, China). l-Lactide (l-LA) and glycolide (GA) were obtained from Changchun SinoBiomaterials Co., Ltd. (Changchun, China) and recrystallized from ethyl acetate under nitrogen atmosphere before use. A PLGA–PEG–PLGA triblock copolymer was synthesized through the ring-opening polymerization (ROP) of l-LA and GA with PEG as a macroinitiator and Sn(Oct)_2_ as a catalyst. The *M*_n_s of PEG and PLGA were 1350 and 1500 g·mol^−1^, respectively. In addition, the molar ratio of l-LA and GA in PLGA segment is 75:25. Both *M*_n_ and molar ratio were calculated from proton nuclear magnetic resonance (^1^H NMR) spectrum, which was recorded on a Bruker AV 300 NMR spectrometer (Bruker Corporation, Billerica, MA, USA) in chloroform-*d* (CDCl_3_).

### 2.4. Phase Diagram and Rheology Analysis of Thermogel

The sol–gel transition behavior of PLGA–PEG–PLGA copolymer in phosphate-buffered saline (PBS) was tested by a vial inverting test with a temperature increment of 1 °C per 5 min. The critical gelation temperature (CGT) was recorded when no visible flow was observed within 30 s after vertically inverting the vial.

The rheological study of PLGA–PEG–PLGA triblock copolymer solution in PBS of pH 7.4 with the optimized concentration of 20 wt % was conducted on a MCR 302 rheometer (Anton Paar, Graz, Austria). The test temperature was set to increase from 15 to 50 °C at a speed of 0.5 °C·min^−1^. The storage modulus (*G*’) was detected under a controlled strain of 1% and a frequency of 10 rad·s^−1^. Besides, the changes of *G*’ of thermogel at 37 °C over time were also tested.

### 2.5. Preparation of PCL/Gel Composite Scaffold

The PCL/Gel composite scaffold was fabricated by impregnating the PCL scaffold in the aqueous solution of PLGA–PEG–PLGA copolymer. Briefly, the PCL scaffold was immerged into a 75% ethanol for 2 h, and then rinsed with PBS (pH 7.4) for 1 h. The PLGA–PEG–PLGA copolymer at a concentration of 20 wt % was dissolved in PBS at 4 °C, and then dropped onto the surface of PCL scaffold in a 0.5 mL Eppendorf (EP) tube. The EP tube was then sent for centrifugation to obtain a full penetration of thermogel into the scaffold pore as reported previously [12]. The scaffold was then removed from the tube and incubated at 37 °C for 15 min to initiate gelation.

### 2.6. Mechanical Properties of Scaffolds

Rabbit osteochondral (OC) plug was obtained from distal femoral condyles. Unconfined compression tests were performed on the cylindrical sample of PCL scaffold and PCL/Gel composite scaffold with a method similar to the previous protocol [13]. The height and diameter of each sample were recorded for later calculation. The sample was subjected to a stress relaxation test to obtain the stress-strain curves. Samples (*n* = 4) were loaded with a force of 0.02 N until the load cell plate came into contact with the sample. After equilibrium was achieved, stress relaxation test was conducted with a compressive deformation of 0.06 mm·min^−1^ to 10% of height of the sample. Samples were then released to reach equilibrium of displacement (1200 s).

### 2.7. Isolation and Culture of BMMSCs

The isolation, culture, and identification of BMMSCs were performed according to the previous reports in our group [14,15]. BMMSCs reached 80% to 90% confluence, and were trypsinized with 0.25% trypsin/0.1% ethylenediaminetetra-acetic acid (EDTA) for subculture at 1:2. BMMSCs at Passage 5 or 6 were used for further experiments.

### 2.8. Cell Seeding and Cells–Scaffold Culture

The BMMSC pellet containing 5.0 × 10^5^ cells was mixed with 40.0 μL of PLGA–PEG–PLGA solution at 4 °C. Then the mixed cells–copolymer solution was incorporated into the porous PCL scaffold and incubated at 37 °C for 15 min to form steady hydrogel as aforementioned. The same volume of BMMSCs-embedded thermogels was dripped into a 96-well plate as a control. In addition, 40.0 μL of BMMSCs suspension with the same cell concentration was seeded onto the PCL scaffold using centrifugation method mentioned before [12] and then incubated for 15 min at 37 °C and 5% (*V*/*V*) CO_2_ atmosphere for initial attachment. The exudative cell suspension was collected and reloaded onto the scaffold. The cells-seeded PCL scaffold and PCL/Gel composite scaffold were transferred to 24-well plates designed for suspension culture and kept in a 37 °C incubator for 2 h to allow further cell attachment before adding 2.0 mL of fresh MEM with alpha modification (α-MEM) supplemented with 10% (*V*/*V*) fetal bovine serum (FBS; Gemini BioProducts, Woodland, CA, USA), and 1% penicillin and streptomycin (Invitrogen, Carlsbad, CA, USA).

For cell proliferation assay and DNA content analysis, the cells-seeded scaffolds were cultured for one week in growth medium, *i.e.*, α-MEM. For chondrogenesis analysis, the cells-seeded constructs were cultured in chondrogenic differentiation medium (RASMX-90041; Cyagen Biosciences Inc., Guangzhou, China) after three days of culture in growth medium. The culture medium was changed every two days.

### 2.9. Cell Viability and Proliferation in Scaffolds

Cell viability assessment in scaffolds was determined using a LIVE/DEAD Viability/Cytotoxicity assay (Invitrogen, Carlsbad, CA, USA) under Leica TCS-SP8 confocal laser microscopy (CFLM; Leica, Nussloch, Germany). The cells-seeded PCL scaffold and PCL/Gel composite were cultured in growth medium for 72 h. Then the cells–scaffold constructs (*n* = 3) were washed in PBS at pH 7.4, three times, followed by the incubation in 4% (*W*/*V*) paraformaldehyde for 30 min. Each construct was immersed in 500.0 μL of PBS with 2.0 mM calcein AM and 4.0 mM ethidium homodimer-1 reagents, and incubated for 2 h at 37 °C. Excitation wavelength of 568 or 488 nm was used to detect the fluorescence of calcein AM (live cells = green) or ethidium homodimer-1 (dead cells = red). Non-seeded scaffolds were also stained as blank control to avoid background effect.

The proliferation activity of cells was quantified on one, seven, or 14 days *in vitro* culture using a Cell Counting Kit-8 assay (CCK-8; Dojindo Laboratories, Kumamoto, Japan) according to the manufacturers’ instructions. Briefly, cells-seeded scaffolds (*n* = 3) were gently rinsed in PBS and then submerged in a mixed solution of 10.0 μL of CCK-8 reagent with 90.0 μL of fresh medium at 37 °C for 2 h. The absorbance readings at 450 nm were observed using a plate reader. The cell content was normalized with standard curve of BMMSC proliferation.

### 2.10. Biochemical Analyses

For biochemical analyses (*n* = 3), specimens were digested in a pre-prepared papain solution containing 0.5 M EDTA, 0.05 M cysteine hydrochloride, and 1.0 mg·mL^−1^ papain enzyme (Sigma, St. Louis, MO, USA) at 60 °C for 12 h. The aliquots of the sample digestion were used for the measurements of DNA and proteoglycan contents. DNA content was measured using a fluorescence assay. Sample digestion was kept at 37 °C for 20 min with 200.0 μL of Hoechst 33258 working solution at a concentration of 2.0 μg·mL^−1^. The fluorescence was read at 360 nm for excitation and 460 nm for emission. The DNA content was normalized with a standard curve of calf thymus DNA (Sigma, St Louis, MO, USA). Total glycosaminoglycan (GAG) content was determined using a 1,9-dimethylmethylene blue (DMMB; Sigma, St. Louis, MO, USA) dye-binding assay with chondroitin-6-sulfate from shark (Sigma, St. Louis, MO, USA) as a standard. Briefly, 20.0 μL of sample was mixed with 200.0 μL of DMMB reagent, and absorbance was read at 525 nm.

### 2.11. Cartilage-Specific Gene Expression Analyses

At pre-designated time points, samples (*n* = 3) were homogenized in Trizol Reagent (Invitrogen, Carlsbad, CA, USA) with a tissue grinder, and RNA was extracted according to the manufacturer's instruction. Concentration of the isolated RNA was determined by an ND-1000 spectrophotometer (Nanodrop Technologies, Thermo Scientific, Carlsbad, CA, USA). One microgram of RNA from each sample was reversely transcribed into cDNA using MMLV Reverse Kit (Promega, Madison, WI, USA), and real-time reverse transcription polymerase chain reaction (RT-PCR) analysis was performed using ABI 7300 real-time PCR system (Applied Biosystems, Foster City, CA, USA) with SYBR Green PCR Master Mix (Toyobo, Osaka, Japan).

The relative gene expression was expressed by fold difference that was calculated as 2^ΔΔ*C*T^. The relative expression changes in these target genes were quantified by normalizing their expression to that of housekeeping gene glyceraldehyde-3-phosphate dehydrogenase (*GAPDH*). Relative quantification of genes expression was given as percentage of the *GAPDH* product. The PCR primers are listed in Table 1.

### 2.12. Statistical Analyses

All data were expressed as means ± standard deviation (SD) and represented at least three independent experiments. Datum analysis was performed using PASW Statistics 18.0 software (SPSS Inc., Chicago, IL, USA). All data were analyzed using a two-way ANOVA test. *p* < 0.05 was considered statistically significant, and *p* < 0.01 and *p* < 0.001 were considered highly significant. When ANOVA results were significant, post-hoc analysis was performed *via* Tukey’s multiple comparison test. All analyses were carried out using GraphPad Prism version 6.0 for Windows (GraphPad Software, San Diego, CA, USA).

## 3. Results

### 3.1. Fabrication and Characterization of PCL Scaffold

**Figure 2:**
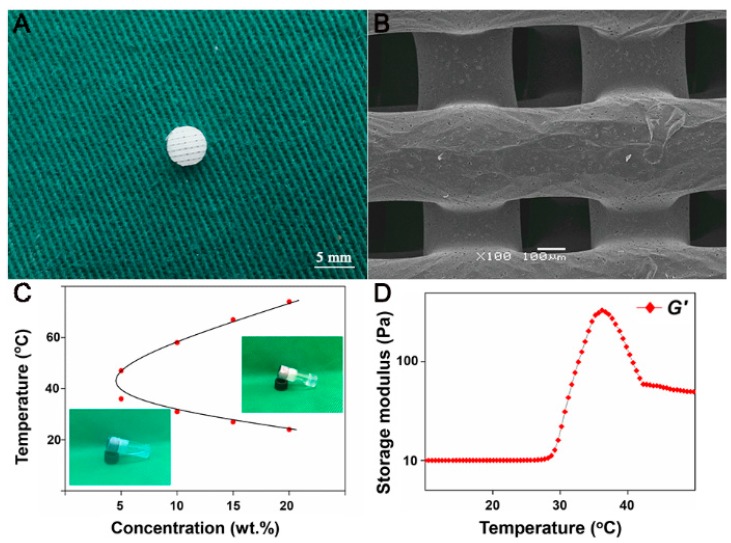
Characterization of PCL scaffold and PLGA–PEG–PLGA thermogel. Gross observation (**A**) and SEM image (**B**) of PCL scaffold fabricated by FDM. Sol-gel phase diagrams of PLGA–PEG–PLGA copolymer in PBS with different concentrations (**C**). Rheological study of PLGA–PEG–PLGA copolymer solution at a concentration of 20 wt % (**D**).

The PCL scaffold fabricated by FDM measuring 10 × 10 × 2 mm^3^ was trimmed into cylindrical samples with a 5-mm diameter corneal trephine. The resultant sample with 2.0 mm thickness and 5.0 mm in diameter was shown in Figure 2A. As revealed by SEM, the PCL scaffold showed homogenously porous structure with highly interconnected pores, whose sizes ranged from 280.3–320.1 μm (Figure 2B). The mean pore sizes of PCL scaffold were 303.3 ± 32.7 μm, and mean porosity of the scaffold was 65% ± 0.07%.

### 3.2. Assessments of PLGA–PEG–PLGA Thermogel

The sol-gel phase diagram of PLGA–PEG–PLGA copolymer in PBS was shown in Figure 2C. The CGT of copolymer in PBS (20 wt %) was 30 °C, which was around body temperature (*i.e.*, 37 °C) and appeared as an appropriate working temperature. Therefore, the copolymer solution with a concentration of 20 wt % was suitable for potential clinical application. This concentration of thermogel was used in the subsequent experiments of this study.

Rheological test was performed to assess the change of *G′ versus* the increase of temperature. As the temperature increased from 10 to 37 °C, the *G′* of the thermogel increased to 425.0 Pa (Figure 2D).

### 3.3. Mechanical Properties of Scaffolds

**Figure 3:**
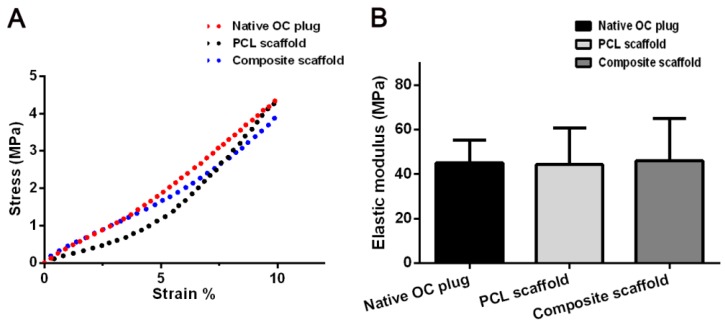
Compressive strength (**A**) and elastic moduli (**B**) of PCL scaffold, composite scaffold, and native OC plug. Results are expressed as mean ± SD (*n* = 3).

Compression measurements were performed to compare the compressive stiffness among PCL scaffold, composite scaffold, and native OC plugs. Figure 3 displays the stress–strain curves of all the tested groups. The elastic modulus was determined by the applied force normalized to the sample cross-sectional area divided by the compressive strain, which could be calculated as the ratio of stress to strain, *i.e.*, the slope of the stress–strain curve for each sample.

Thermogel displayed the weakest and almost negligible mechanical strength compared to other scaffolds and native OC plug (data not shown). As shown in Figure 3, no differences of compressive strength and elastic moduli were revealed among PCL scaffold, composite scaffold, and OC plugs (*p* > 0.05).

### 3.4. Cell Viability and Proliferation

**Figure 4:**
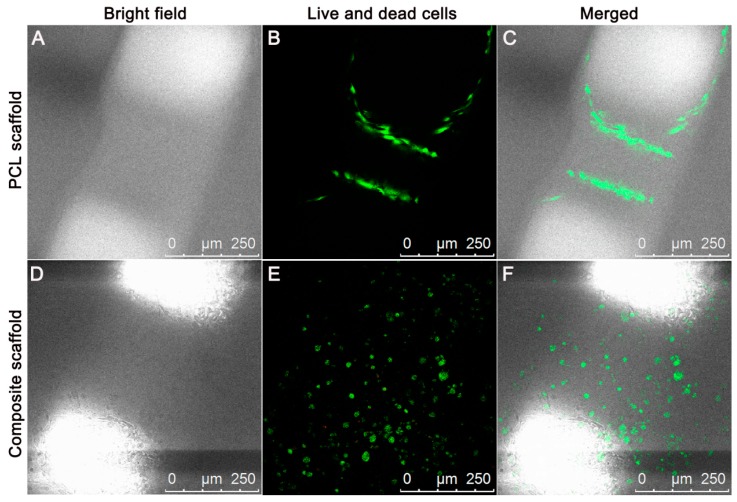

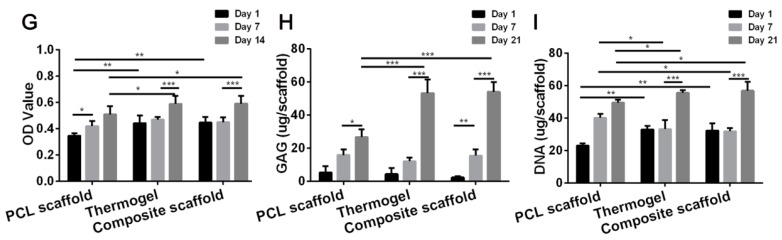
Representative images of attachment, viability, and distribution of BMMSCs in PCL scaffold (**A**–**C**) and composite scaffold (**D**–**F**). Bright field views show the contours of the PCL scaffold and composite (**A**,**D**). The dark area indicates the PCL fibers, and the bright area is the scaffold pores. CFLM images of LIVE/DEAD staining demonstrated *in vitro* cell viability and proliferation of three groups after culture for 72 h (**B**,**E**). The distribution of BMMSCs on the PCL scaffold and PCL/Gel composite scaffold were shown in the merged images (**C**,**F**). (Red, dead cells; green, live cells; scale bar = 250 μm). CCK-8 assay and DNA content showed that the increased number of cells in the three groups over time (**G**,**H**). GAG deposition in various scaffolds by embedded BMMSCs (**I**). Results are expressed as mean ± SD (*n* = 3; * *p* < 0.05, ** *p* < 0.01, *** *p* < 0.001).

After culture in growth medium for 72 h, the LIVE/DEAD assay showed that BMMSCs survived well in both the PCL scaffold and composite with minimal dead cells (Figure 4A–F). BMMSCs displayed polygonal or elongated shapes in the PCL scaffold, while cells encapsulated in the PCL/Gel composite scaffold assumed a chondrocyte-like round morphology. Moreover, BMMSCs seeded in the PCL scaffold mainly attached on the PCL fibers with no cells observed in the pores of scaffold. However, BMMSCs embedded in the composite scaffold exhibited an evenly distribution fashion with round chondrocyte-like cells filled in the pores. These findings suggest excellent compatibility of PCL scaffold or PLGA–PEG–PLGA thermogel toward BMMSCs. Additionally, compared to the PCL scaffold, the composite scaffold filled with thermogel seemed to provide a suitable growth microenvironment closer to cartilage, which was confirmed by cell morphology and distribution.

CCK-8 assay demonstrated that all the cells–scaffold constructs showed an increasing proliferation during 14 days of *in vitro* culture (Figure 4G). However, the number of cells in thermogel and thermogel-filled scaffold on Day 7 did not increased significantly compared to that on Day 1 (*p* > 0.05). On the contrary, cells in the PCL scaffold continued to proliferate after 1 day. Notably, the number of MSCs in the composite scaffold markedly increased and surpassed that in the PCL scaffold at 14 days.

DNA contents of all the cells-seeded scaffold groups significantly increased after 7 days’ culture, which was in accordance to the proliferation trend observed by CCK assay. In addition, DNA contents of the thermogel and composite scaffold groups reached a higher level at three weeks of *in vitro* culture, compared to those of PCL scaffold (Figure 4H).

### 3.5. Cartilaginous Matrix Production on Scaffolds In Vitro

GAG content was detected to quantify cartilaginous matrix production by constructs. All groups showed continuously increasing GAG contents after cultured in chondrogenic medium for 7 days (Figure 4I). On Day 21, significantly higher GAG contents were found in thermogel and composite scaffold groups than those of the PCL scaffold group.

### 3.6. Cartilage-Specific Gene Expression Analyses

To compare the chondrogenic capacity of seeded BMMSCs among PCL scaffold, thermogel, and composite scaffold, we measured gene expressions of type I collagen (*Col I*), type II collagen (*Col II*), aggrecan (*AGC*), and alkaline phosphatase (*ALP*).

**Figure 5:**
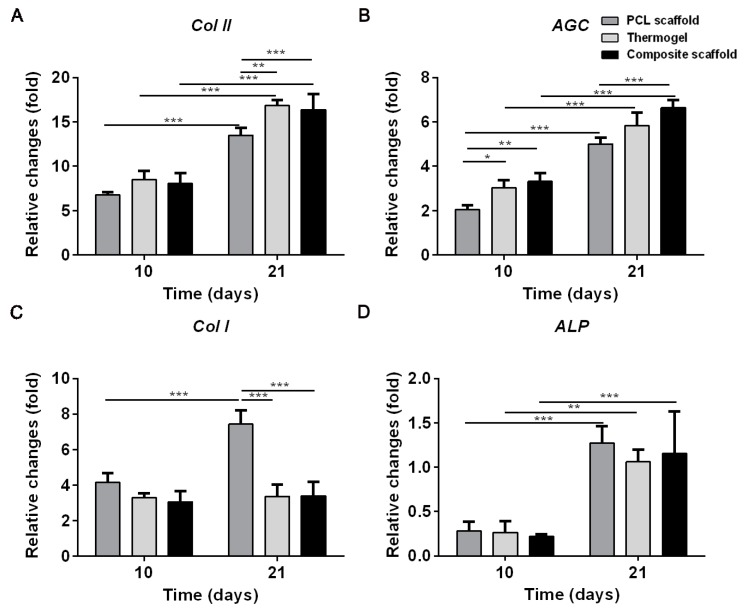
Expression of cartilage-specific genes of *Col II* (**A**) and *AGC* (**B**), fibrous-cartilage gene of *Col I* (**C**), and the osteogenesis marker gene of *ALP* (**D**) of BMMSCs within various scaffolds. Results are expressed as mean ± SD (*n* = 3; * *p* < 0.05, ** *p* < 0.01, *** *p* < 0.001).

Significant differences in gene expression were found among scaffolds at 10 and 21 days of culture (Figure 5). Greater upregulation of hyaline-cartilage specific genes *Col II* and *AGC* as well as osteogenesis marker *ALP* were detected in all three groups of cells–scaffold constructs at 21 days compared to those at 10 days. Notably, increased expression of fibrocartilage-related marker *Col I* was only found in the PCL scaffold after 21 days of culture.

In addition, the gene expression differed among all three test groups. There appeared to be a trend that the expression of *Col II* and *AGC* in the thermogel and composite scaffold groups were higher than those in the PCL scaffold at 10 days. However, the gene expressions of *Col II,* *Col I,* and *ALP* were similar among all three groups of scaffolds, whereas *AGC* expression levels in the thermogel or thermogel-containing composite scaffold groups were greater than those in the PCL scaffold groups. On Day 21, the *Col II* and *AGC* expression levels were highest in the thermogel and composite scaffold groups, while the highest amount of *Col I* was found in the PCL scaffold group. The *ALP* expression level was similar among all three scaffold groups.

## 4. Discussion

Scaffolds, growth factors, and cells are three building blocks for cartilage tissue engineering. An ideal scaffold for cartilage tissue engineering must be biocompatible with its degradation products doing no harm to host tissues and biomimetic, so that the seeded cells or native host cells can colonize and grow. For scaffold implantation in a weight-bearing joint, the scaffold should possess adequate mechanical strength to withstand the mechanical condition. Recently, with the assistance of FDM, we designed and fabricated a bioabsorbable PCL scaffold with fully-interconnected pores and excellent mechanical integrity. As shown in compression tests, the porous PCL scaffold has an elastic modulus similar to rabbit native OC tissue. However, in addition to the mechanical properties, it is indispensable that the scaffold should facilitate cell adhesion, growth, and even chondrogenesis. As other solid scaffolds, the PCL scaffold turned out to be a sub-optimal structure for cell migration and spatial organization, which may be attributed to the material property itself and the scaffold architectures, such as porosity and size [16,17,18]. A functional scaffold with adequate mechanical strength and suitable interfaces for cell adhesion and proliferation is expected to meet these challenging demands.

Hydrogels have been used as a soft scaffold or cell carrier for cartilage repair because they contain a large proportion of water and allow easy exchange of nutrients and metabolic waste with surrounding tissues and the capacity of maintaining chondrocyte phenotype [6,19]. Among various hydrogels, the thermo-sensitive hydrogel from PLGA–PEG–PLGA copolymer has been paid much attention recently due to its advantages, such as ease of use, efficiency of cell encapsulation, and drug delivery [20,21,22]. However, hydrogels are inherently too weak to play the mechanical roles in joint cartilage repair, owing to their inferior mechanical property, which has restricted further *in vivo* application.

In the current work, the PLGA–PEG–PLGA thermogel was incorporated into the porous PCL scaffold. This combination of biomaterials can fulfill demanding needs of scaffold materials in cartilage tissue engineering. The PLGA–PEG–PLGA copolymer was synthesized, and the chemical structure and composition of copolymer was confirmed by ^1^H NMR. The sol-gel phase diagram of PLGA–PEG–PLGA copolymer (20 wt %) in PBS was revealed. The copolymer maintained a sol state at 4 °C, which is convenient for cell encapsulation, and transformed into hydrogel when temperature increased to 37 °C. The range of temperature from 4 to 37 °C is favorable for thermogel manipulation in the setting of clinical application.

The addition of PCL scaffold to thermogel system greatly increases the mechanical property of the thermogel construct alone. The PCL/Gel scaffold obtained an elastic modulus similar to rabbit native OC tissue as shown in Figure 2, the thermogel in such composite scaffold provided a beneficial microenvironment for cell distribution and survival, which was confirmed by LIVE-DEAD assay after three days of *in vitro* culture. Notably, the encapsulated cells in the composite scaffold exhibited a round shape. In addition, these round MSCs reside evenly within the thermogel filled in the pores and coated on the surface of PCL scaffold. In contrast, it is not surprising that we found a limited amount of cells attaching to the PCL scaffold, which may be unfavorable for tissue engineering. This is due to the hydrophobic property of PCL scaffold and scaffold architecture [5]. In our study, a relative large pore size of 300 μm is used for PCL scaffold fabrication, which evidently facilitates thermogel penetration and exchange of nutrient and gas, as well as in-growth of cells and extracellular matrix. These findings were also consistent with the results of CCK assay, which revealed the active cell proliferation in all three kinds of scaffolds. However, there seemed to be an initial faster cell growth in the PCL scaffold compared to that in the thermogel or composite scaffold. This may be attributed to the rapid cell attachment and proliferation on the outer surface of PCL scaffold such as on the fibers and fiber junctions of PCL scaffold. Meanwhile the cell aggregation on the outer surface will subsequently restrict cell penetration into the center of the scaffolds [23]. Therefore, the composite scaffold exhibited a biomimetic microenvironment allowing for better cell retention and proliferation, and matrix deposition, while providing enhanced mechanical strength for *in vivo* application.

To evaluate the chondrogenic ability of seeded BMMSCs in thermogel, porous PCL scaffold, and composite scaffold, we analyzed the matrix production of the cells-seeded scaffold cultured in chondrogenic medium for up to three weeks. GAG was higher in the PCL scaffold group on Day 1 and 7. It might be due to the initially slow cell proliferation in thermogel observed by CCK assay. However, sustaining cell proliferation and matrix production were found after BMMSCs colonizing in thermogel for one week, which was evidenced by more abundant GAG produced in the thermogel group and composite scaffold groups at three weeks. The phenomenon might be due to cells acclimatizing to microenvironment after changing from a monolayer culture to a 3D condition [24].

Although AGC expressions were lowest in the PCL scaffold, similar levels of *Col II*, *Col I*, and *ALP* gene expression were found in all three types of scaffolds at 10 days of chondrogenic induction. After 21 days of *in vitro* chondrogenic culture, the encapsulated BMMSCs in thermogel and thermogel-containing composite scaffold exhibited a similarly higher amount of cartilage-specific gene expression (e.g., *Col II* and *AGC*) compared to cells seeded on the PCL scaffold. The secretion of ECM products, such as GAG and *Col II,* evidenced that BMMSCs seeded in thermogel and composite scaffold possessed the greatest chondrogenic capacity, which may be attributed to the favorable growth and pro-chondrogenic microenvironment of thermogel. Interestingly, BMMSCs seeded on the PCL scaffold showed stronger *Col I* and *ALP* expression throughout the three weeks chondrogenic induction compared to those in thermogel and composite scaffold. The BMMSCs on the PCL scaffold took on an elongated shape like fibroblasts, while the round chondrocyte-like BMMSCs in the thermogel have contributed to the decreased level of osteogenesis and dedifferentiation marker of chondrocytes, that is, *Col I* and *ALP*. It has also been confirmed by previous studies, which showed that the cell morphology had an impact on cell differentiation [25,26].

The addition of PCL scaffold to thermogel system greatly increases the mechanical property of thermogel construct alone. The PCL/Gel scaffold obtained an elastic modulus (45.3 ± 16.5 MPa) similar to rabbit native OC tissue (45.1 ± 10.3 MPa) as shown in Figure 2. The elastic modulus of the composite scaffold was not compared to that of normal human OC plug, which was unavailable in current literature. However, the elastic modulus of PCL/Gel scaffold has surpassed that of normal human cartilage in patellar groove (0.5 ± 0.1 MPa) in a previous study [27], indicating a potential clinical application.

Although some meaningful findings were revealed, the present study has some limitations. Firstly, investigation on the influence of pore sizes on overall mechanical strength and cell retaining capacity of composite scaffold has not been performed. However, we chose the pore size based on an elaborated research, which reported that scaffolds with a pore size of 300 μm stimulated significantly higher cell proliferation, chondrogenic gene expression, and matrix deposition compared to scaffolds with smaller mean pore sizes of 94 and 130 μm [16]. Second, the current work is a preliminary study mainly focused on *in vitro* applicability of 3D PCL/Gel composite scaffolds, the future studies will be conducted on animals, such as rabbits.

## 5. Conclusions

Taken together, a practical 3D composite scaffold composed of a PCL backbone network and PLGA–PEG–PLGA thermogel was fabricated, which possessed significantly enhanced mechanical strength and a suitable biocompatible microenvironment for cell growth and cartilage differentiation. This advanced scaffold with the combination of rigid and soft constituents is expected to be promising for cartilage and OC defect repair.

## Figures and Tables

**Table 1:** Primer sequences used for real-time PCR.

Gene	Forward primers (5′-3′)	Reverse primers (5′-3′)
*Col I*	TGGCAAGAACGGAGATGACG	GCACCATCCAAACCACTGAA
*Col II*	CCACGCTCAAGTCCCTCAAC	AGTCACCGCTCTTCCACTCG
*ACG*	CGTGGTCTGGACAGGTGCTA	GGTTGGGGTAGAGGTAGACG
*ALP*	CGACACGGACAAGAAACCCT	TGTTGTGAGCGTAGTCCACC
*GAPDH*	CCATCACCATCTTCCAGGAG	GATGATGACCCTTTTGGCTC

*Col I*: type I collagen; *Col II*: type II collagen; *AGC*: aggrecan; *ALP*: alkaline phosphatase; *GAPDH*: glyceraldehyde-3-phosphate dehydrogenase.

## Abbreviations

The following abbreviations are used in this manuscript:

ALP	Alkaline phosphatase
CFLM	Confocal laser microscopy
CCK-8	Cell Counting Kit-8 assay
Col I	Type I collagen
Col II	Type II collagen
CGT	Critical gelation temperature
DMMB	1,9-dimethylmethylene blue
ECM	Extracellular matrix
EDTA	Ethylenediaminetetra-acetic acid
EP	Eppendorf
FBS	Fetal bovine serum
FDM	Fused deposition modeling
GAPDH	Glyceraldehyde-3-phosphate dehydrogenase
GAG	Glycosaminoglycan
MSCs	Mesenchymal stromal cells
α-MEM	MEM with alpha modification
MP	Melting point
Mn	Number-average molecular weight
OC	Osteochondral
1H NMR	Proton nuclear magnetic resonance
PLGA–PEG–PLGA	Poly(lactide-*co*-glycolide)-*block*-poly(ethylene glycol)-*block*-poly(lactide-*co*-glycolide)
PCL	Poly(ε-caprolactone)
PBS	Phosphate-buffered saline
ROP	Ring-opening polymerization
SEM	Scanning electron microscopy
SD	Standard deviation
Sn(Oct)2	Stannous octoate
3D	Three-dimensional
